# Visual neglect in posterior cortical atrophy

**DOI:** 10.1186/1471-2377-10-68

**Published:** 2010-08-10

**Authors:** Katia Andrade, Dalila Samri, Marie Sarazin, Leonardo C de Souza, Laurent Cohen, Michel Thiebaut de Schotten, Bruno Dubois, Paolo Bartolomeo

**Affiliations:** 1 INSERM UMR_S 975, Centre de Recherche de l'Institut du Cerveau et de la Moelle épinière, Cognition, neuro-imagerie et maladies du cerveau, Paris, France; 2 Université Pierre et Marie Curie, Paris 6, Paris, France; 3 Fédération de Neurologie, Hôpital Pitié-Salpêtrière, AP-HP, Paris, France; 4 INSERM UMR_S 975, Centre de Recherche de l'Institut du Cerveau et de la Moelle épinière, Neuropsychologie et neuroimagerie, Paris, France; 5 Natbrainlab, Section of Brain Maturation, Institute of Psychiatry, King's College London, London, UK; 6 Department of Psychology, Catholic University, Milan, Italy

## Abstract

**Background:**

In posterior cortical atrophy (PCA), there is a progressive impairment of high-level visual functions and parietal damage, which might predict the occurrence of visual neglect. However, neglect may pass undetected if not assessed with specific tests, and might therefore be underestimated in PCA. In this prospective study, we aimed at establishing the side, the frequency and the severity of visual neglect, visual extinction, and primary visual field defects in an unselected sample of PCA patients.

**Methods:**

Twenty-four right-handed PCA patients underwent a standardized battery of neglect tests. Visual fields were examined clinically by the confrontation method.

**Results:**

Sixteen of the 24 patients (66%) had signs of visual neglect on at least one test, and fourteen (58%) also had visual extinction or hemianopia. Five patients (21%) had neither neglect nor visual field defects. As expected, left-sided neglect was more severe than right-sided neglect. However, right-sided neglect resulted more frequently in this population (29%) than in previous studies on focal brain lesions.

**Conclusion:**

When assessed with specific visuospatial tests, visual neglect is frequent in patients with PCA. Diagnosis of neglect is important because of its negative impact on daily activities. Clinicians should consider the routine use of neglect tests to screen patients with high-level visual deficits. The relatively high frequency of right-sided neglect in neurodegenerative patients supports the hypothesis that bilateral brain damage is necessary for right-sided neglect signs to occur, perhaps because of the presence in the right hemisphere of crucial structures whose damage contributes to neglect.

## Background

Posterior cortical atrophy (PCA) is a rare, early-onset neurodegenerative disease, characterized by a progressive impairment of higher order visual functions out of proportion to other cognitive disabilities [1] and occipito-parietal damage, which is often more severe in the right hemisphere [2,3]. Asymmetric parietal damage might predict a frequent occurrence of visual neglect and related disorders such as visual extinction in PCA patients. Despite this, neglect and extinction appear to be relatively rare findings in PCA [4,5] mainly observed late in the course of the disease [1]. However, neglect may easily pass undetected if not assessed with specific tests [6]. Thus, a study employing specific neglect tests [7] revealed signs of left-sided neglect in six patients, and of right-sided neglect in one patient out of a group of 15.

Patients with visual neglect are impaired in responding to events occurring on the side opposite to a brain lesion [8,9], mainly affecting the right temporo-parietal region and its connections with the frontal lobe [10,11]. Therefore, in stroke patients left-sided neglect is more frequent and severe than right-sided neglect [12]. Patients with left brain damage may also show signs of right-sided neglect, albeit more rarely and in a less severe form [12]. Concomitant damage to the right hemisphere might be important for the emergence of right-sided neglect [13]. Neglect often co-occurs with visual extinction, the failure to detect contralesional stimuli on bilateral presentation with preserved detection of the same stimuli when presented in isolation[14] or with primary visual field defects, such as homonymous hemianopia[15]. Diagnosis is important, because neglect has a dramatic impact on patients' functional disability [16], requires specific rehabilitation [17] and increases family burden [16].

In this prospective study, we aimed at establishing the side, the frequency and the severity of visual neglect, visual extinction, and primary visual field defects in an unselected sample of 24 PCA patients, by using standardized visuospatial tests [18].

## Methods

### Subjects

**Figure 1:**
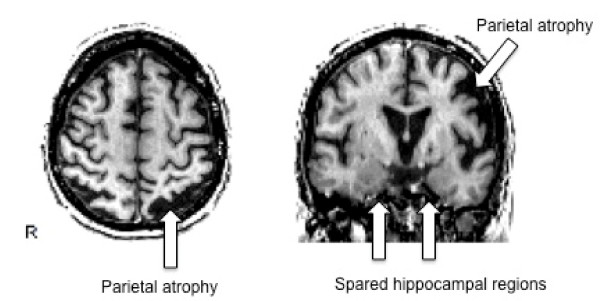
**Brain MRI scan of patient 10**. Axial and coronal T1-weighted MRI scans of patient 10 (see Table 1), showing a pattern of cortical atrophy more pronounced in the occipito-parietal regions (left > right). Note the relative sparing of hippocampal formations.

Twenty-four right-handed patients (18 women), who met the clinical diagnostic criteria of PCA [7], participated in the study. The research protocol was approved by the local ethical committee for clinical research and all procedures involving the participant were conducted according to institutional guidelines in compliance with the regulations. Informed consents were obtained from the patients or their families. Average age at onset was 57.66 years (range 48-74). Patients underwent a basic neurological examination and a full battery of neuropsychological tests, including standard cognitive tests and tests designated to assess dysfunctions of the dorsal and ventral cortical visual streams, 4.58 years on average after symptom onset. As expected, patients presented prominent visuoperceptive and visuospatial disorders, as well as important attentional deficits, while episodic memory appeared less impaired (see Table 1). The average MMSE score was 19.00 (range 8-27). Brain MRIs were acquired for clinical reasons. On visual inspection, all patients had a predominant posterior and bilateral pattern of atrophy (Fig. 1), in the absence of focal brain lesions. The available independent reports of experimented neuro-radiologists confirmed this topography.

**Table 1:** Patients' demographical and neuropsychological data.

Patient	Sex/Age/Education level	Years since symptom onset	MMSE	Episodic memory impairment	Attentional deficits	Elements of Balint's syndrome	Elements of Gerstmann's syndrome	Visual agnosia	Reading impairment	Apraxia
1	F/58/3	2	23	+	+ +	+ +	-	-	-	CA, IA
2	F/69/1	2	16	+	+ +	Sm	Ac	-	+	CA, IA
3	F/70/2	9	21	+	+ +	Sm, OA	Ac, RLc	-	?	CA
4	F/60/2	2	26	-	+	Sm	-	-	+/-	CA, IA
5	M/60/3	6	24	+	+ +	Sm, OA	RLc, FA	-	+	CA
6	F/53/3	3	13	+	+ +	+ +	Ac, Ag	?	+	CA, IA
7	F/59/1	2	14	+	+ +	Sm, OA	Ac, Ag, FA	-	+	CA, IA
8	M/77/1	4	21	+/-	+	Sm	+ +	-	-	CA, IA
9	F/59/1	10	8	+	+ +	+ +	+ +	+	+	CA, IA
10	F/63/3	4	24	-	+ +	+ +	Ag	-	-	CA, IA
11	F/82/3	8	19	-	+	+ +	Ag	-	+	CA, IA
12	F/56/3	2	20	+	+ +	Sm	+ +	-	-	CA, IA
13	F/61/2	6	14	+/-	+ +	+ +	+ +	+ +	++	CA, IA
14	F/59/1	4	13	+	+ +	+ +	+ +	+	+	CA, IA
15	M/64/3	6	20	+	+ +	+ +	+ +	-	+	CA, IA
16	F/57/1	5	15	+	+ +	Sm	+ +	-	+	CA, IA
17	F/73/3	8	22	-	+	Sm	Ag	+	+	CA
18	F/63/1	4	21	+/-	+ +	Sm	Ac, Ag	-	-	CA, IA
19	F/55/1	3	18	+	+ +	Sm	+ +	-	+	CA, IA
20	F/53/3	5	19	+/-	+ +	+ +	+ +	-	+	CA, IA
21	F/57/3	2	22	-	+ +	+ +	Ac, Ag	-	+	CA, IA
22	M/65/3	6	18	+	+ +	-	Ac, Ag, RLc	-	-	CA, IA
23	M/56/3	2	27	-	+	Sm	RLc	-	+	CA, IA
24	M/67/3	5	18	+	+	Sm	FA	+/-	-	IA

Education level: level 1, < 9 years of schooling; level 2, 9-12 years, level 3, >12 years. MMSE, Mini mental state examination; +, present; ++, severe or complete syndrome; +/-, mild; -, absent; ?, unavailable data; Sm, simultanagnosia; OA, optic ataxia; RLc, right-left confusion; Ag, agraphia; Ac, acalculia; FA, finger agnosia; CA, constructive apraxia; IA, ideomotor apraxia.

### Procedure

An expert clinical neuropsychologist (DS) administered the tests and ensured homogeneity of testing conditions and of scoring. Patients were tested in a quiet environment.

The examiner sat in front of the patient and presented the test material centered on the patients' body midline.

### Neglect examination

Line bisection [18]. Patients were asked to mark the middle of five 20-cm long and 1-mm wide lines. The lines were presented separately, each centered on an A4 horizontal sheet. Deviation from the true center was measured to the nearest millimeter, with a positive sign for rightward deviations, and a negative sign for leftward deviations. The cumulated percentage of deviation from the true centre for all the lines was calculated. Bells test [19]. Subjects were asked to circle 35 targets (black ink drawings of bells), presented on a horizontal A4 paper sheet, along with 280 distracters, which were equally distributed in seven columns. The severity of neglect was estimated by using a previously described laterality index [14], which provides a quantitative score of spatial bias that is independent of the overall level of performance. Overlapping figures [20]. Five test stimuli were presented one at a time, each bearing five overlapping figures on a vertical A4 sheet. Each pattern consisted of two figures overlapping on the right and two on the left side of the card, all of them overlapping with a centrally located figure. Patients were asked to name the objects they could detect, but they were not informed of the number of figures in each stimulus. In the present study, however, the overlapping figures test was used only as an ancillary source of evidence about patients' visuospatial processing abilities and not for diagnosis of neglect, because of its sensitivity to simultanagnosia, which is frequently present in patients with PCA [4].

Performance on paper-and-pencil tests was evaluated against that of a large sample of healthy French subjects from a previous study (n = 456 to 576, depending on the tests) [6]. In this study, control subjects were distributed in four age ranges (20-34 years; 35-49 years; 50-64 years; 65-80 years) and three levels of education (1, < 9 years of schooling; 2, 9-12 years; 3, >12 years). For each test, performance was considered as pathological when the score was lower than the fifth percentile of the control group [6].

In addition to the neglect tasks, patients underwent neuropsychological assessments that were grouped under five broad headings: 1) episodic memory (Grober and Buschke test); 2) attention and working memory (digit spans and Corsi blocks); 3) language and arithmetic (letter fluency, naming, reading and writing; arithmetic's operations); 4) perception (object naming, "cookie thief" scene description, overlapping figures identification); and 5) constructional praxis (spontaneous drawing, copy of geometrical figures and of the Rey figure) and gestural praxis (on imitation and command, uni- and bimanual; object utilization). Patients' cognitive profile is shown in Table 1.

### Visual field examination

Patients' visual field was assessed clinically by wiggling fingers in one or both visual fields. The test consisted of six single unilateral stimuli and six double simultaneous stimuli presented in a pseudorandom order [14]. The examiner controlled central gaze fixation. Lateral homonymous hemianopia was defined as the complete lack of detection of stimuli on one side. Following previous criteria [20], visual extinction was defined as the presence of at least 16% omissions on the same side on double simultaneous stimulation. In the same study, *severe *extinction was defined as the omission of more than 60% of the stimuli on the same side.

## Results

Table 2 reports patients' performance on visual field examination and neglect tests.

**Table 2:** Patients'performance on visuospatial tests.

Patient	DSS L/R hits Max. 12/12	Visual fields	Line bisection (average deviation)	Bell's test L/R hits Max. 15/15	Overlapping figures L/R hits Max. 10/10
1	7/12	LE	+ 19.0*	14/15	9/8
2	7/12	LE	+ 22.0*	9/15*	9/9
3	7/12	LE	+ 14.0*	8/12*	9/8
4	7/12	LE	- 8.4†	15/14	3/5
5	8/12	LE	- 11.6†	14/14	5/8
6	5/12	LE	- 14.6†	14/11†	7/8
7	6/12	LE	- 3.0	7/7	5/3
8	10/12	LE	- 0.2	15/13	10/8
9	U	-	+18.0*	8/7	U
10	0/12	LH	+ 20.4*	12/14	8/7
11	0/12	LH	+ 21.2*	8/13*	1/3
12	0/12	LH	+ 29.0*	12/13	7/7
13	12/6	RE	+ 8.8*	2/1‡	1/0
14	12/7	RE	+ 0.8	10/5†	8/6
15	12/10	RE	- 10.0†	10/10	6/5
16	12/12	normal	- 2.8	14/9†	6/7
17	12/12	normal	+ 3.4	8/11*	2/0
18	12/12	normal	- 8.6†	14/15	10/8
19	12/12	normal	- 8.6#	15/13	7/9
20	12/12	normal	+ 3.6	14/14	9/10
21	12/12	normal	- 0.4	13/13	10/9
22	12/12	normal	- 4.2	15/14	10/10
23	12/12	normal	- 3.8	15/14	9/8
24	12/12	normal	- 2.0	14/14	10/10

DSS, visual double simultaneous stimulation; L, left; R, right; E, extinction; H, homonymous hemianopia. For line bisection, positive values indicate rightwards deviations, negative values leftwards deviations from the true center; *, left-sided neglect; †, right-sided neglect; #, special case (see Table 3 and Results section in the main text); U, unable to perform the task; ‡, the test was discontinued because of a severe impairment in target recognition.

### Visual fields deficits

Three patients (cases 10-12 in Table 2) missed all the left-sided stimuli, thus suggesting the presence of left homonymous hemianopia. Of the remaining patients, eight had mild left extinction (cases 1-8), three showed mild right extinction (cases 13-15), while nine had accurate performance (cases 16-24). Patient 9 could not perform the confrontation test because of severe simultanagnosia.

### Neglect

Overall, sixteen of the 24 patients (66.6%) had signs of visual neglect on at least one test, and fourteen (58.3%) also had visual extinction or hemianopia. The duration of disease in neglect patients ranged from 2 to 10 years. Five patients (20.8%) had neither neglect nor visual field defects. The duration of disease in these patients ranged from 2 to 6 years. The side of neglect was generally consistent with that of visual field deficits, except for 4 patients (cases 4-6 and 13 in Table 2), whose extinction and deviations on line bisection were in opposite directions. Among the patients with neglect, nine had left-sided neglect and seven had right-sided neglect. Left-sided neglect was generally more severe than right-sided neglect. Patients with left-sided neglect and clinical signs of left homonymous hemianopia (cases 10-12) deviated rightwards massively (> 20%) on line bisection, a pattern of performance previously described in stroke patients with such an association of neglect and visual field defect[15]. This association may increase the shift of the subjective center because the contralesional extremity of the line is likely to fall in the visual field deficit, thus further decreasing its contribution to patients' perceptual judgments, a contribution already impaired by the contralesional neglect.

Thirteen patients (cases 1-6, 9-13, 15 and 18) presented neglect on line bisection. These patients deviated consistently towards the same side on the five line bisection trials (see Table 3; for three patients data for individual trials were lost), thus suggesting a systematic lateralized deficit rather than spurious findings resulting from intra-subject variability. Three patients (cases 14, 16 and 17) had neglect signs only on the cancellation task, while four patients (cases 2, 3, 6 and 11) presented neglect on both line bisection and target cancellation (Table 2). Patient 13 had a severe impairment in identifying targets in the cancellation task because of visual agnosia (Table 1). She showed right visual extinction and a mild rightward deviation on line bisection. Patient 19 had a paradoxical performance on line bisection, deviating leftwards in 4 out of five trials, while in the fifth she clearly deviated rightwards. She had accurate performance on the bells test. These patterns of performance make it difficult reaching conclusions about the presence of lateralized deficits in these two patients.

**Table 3:** Detailed data of patients' performance on line bisection.

Patient	1st trial	2nd trial	3rd trial	4th trial	5th trial	Average deviation
1	+ 26	+ 5	+ 22	+ 18	+ 24	+ 19.0*
2	+ 39	+ 27	+ 12	+ 22	+ 10	+ 22.0*
3	+ 15	+ 11	+ 20	+ 3	+ 21	+ 14.0*
4	-	-	-	-	-	- 8.4†
5	-10	-10	-12	-17	- 9	- 11.6†
6	-16	-13	- 20	-15	- 9	- 14.6†
7	-	-	-	-	-	- 3.0
8	0	+ 6	- 5	- 4	+ 2	- 0.2
9	+ 2	+ 19	+ 20	+ 18	+ 31	+18.0*
10	+ 14	+ 28	+ 20	+ 38	+ 2	+ 20.4*
11	+ 18	+ 25	+ 23	+ 23	+ 17	+ 21.2*
12	-	-	-	-	-	+ 29.0*
13	+ 15	+ 12	0	+ 4	+ 13	+ 8.8*
14	- 12	- 12	+ 1	+ 18	+ 9	+ 0.8
15	- 17	- 6	- 8	- 9	- 10	- 10.0†
16	- 3	- 11	- 4	+ 1	+ 3	- 2.8
17	- 9	+ 7	+ 5	+ 7	+ 7	+ 3.4
18	- 12	- 6	- 5	- 10	- 10	- 8.6†
19	- 13	- 32	- 10	- 18	+ 30	- 8.6#
20	+ 9	- 7	+ 3	+ 9	+ 4	+ 3.6
21	+ 3	- 1	- 14	+ 9	+1	- 0.4
22	- 1	- 4	- 4	- 6	- 6	- 4.2
23	- 8	- 2	- 8	- 2	+ 1	- 3.8
24	- 5	+ 2	- 4	- 8	+ 5	- 2.0

*, left-sided neglect; †, right-sided neglect; -, missing data; #, special case (see Results section).

The overlapping figures test was poorly informative about neglect, with patients being frequently unable to identify figures on both sides, as a consequence of their simultanagnosia (Table 1). In some cases, however, the pattern of performance seemed clearly lateralized and was consistent with the outcome of other neglect tests. For example, patients 14 and 18 made more right-sided than left-sided omissions, consistent with their right-sided neglect on target cancellation or line bisection.

We assessed the relationship between neglect severity and variables related to the general evolution of PCA by calculating the correlation coefficients between line bisection deviations and of the bells test laterality index, both in absolute values, and the number of years since symptom onset and MMSE scores. The statistical analysis was performed using the 'R project' software for statistical computing [21].

There were no significant correlations between neglect and disease duration (line bisection, r = 0.04; bells test, r = 0.27; both p > 0.18) and MMSE (line bisection, r = 0.02, p = 0.91), with the exception of a negative correlation between the bells test and the MMSE score (r = -0.41, p = 0.04), which indicates that decreasing MMSE scores correlated with increasing severity of neglect.

## Discussion

Visual neglect can be difficult to assess in PCA, because of its frequent association with deficits of visual perception, such as visual agnosia and simultanagnosia. This may account for previous findings [4,5], based on clinical examination, that neglect rarely occur in PCA. Despite this, visual neglect and visual extinction were frequently observed in the present PCA patients when using specific tests. The use of more extensive neglect batteries [6,22] might further increase the frequency of observation of neglect in PCA. On the other hand, visual neglect may contribute to PCA patients' impaired performance on other tasks implicating a visuospatial component, such as the Corsi block test, text reading, sentence writing and copy of the Rey figure.

Three patients of our series showed clinical signs of left homonymous hemianopia, a very rare finding in neurodegenerative conditions [23]. However, severe neglect may induce lack of responses even for isolated left-sided stimuli, such as those used in the clinical confrontation method, and be mistaken for field loss. This does not seem to be the case for the present patients 10-12, who had severe neglect on line bisection (as typically found in patients with an association of neglect and hemianopia, see ref. 15), but not on the bells test, where they were able to detect between 53% and 80% of left-sided targets (see Table 2). In any case, to confirm the unexpected finding of left hemianopia in PCA, future studies should add visual field perimetry or visual evoked potentials to the standard clinical examination of visual functions.

As in patients with focal lesions [12], left-sided neglect was generally more severe than right-sided neglect. These results are consistent with reports of asymmetries in cortical degeneration in PCA, with the right hemisphere often being more affected than the left hemisphere [2,3]. Non-lateralized deficits of attention and working memory, resulting from injury of right-hemisphere structures like the right inferior parietal lobe [24], may contribute to the presence and severity of neglect. Such structures are commonly damaged in PCA and may also account for the emergence of the neglect syndrome in this neurodegenerative condition. In contrast to evidence coming from stroke patients [12] and from previous results on neglect in PCA [7], right-sided neglect resulted more frequently in PCA than in patients with focal damage to the left hemisphere, especially for line bisection (29% of pathological performance in our sample vs. 6.4% in a previous study [12] using similar stimuli, although a direct comparison is difficult given the differences in sample sizes). Such a finding is in line with evidence that damage of both hemispheres, as expected in PCA [2,3], is more likely to cause signs of right-sided neglect than unilateral damage of the left hemisphere [13], and it is consistent with the hypothesis that damage to the right inferior parietal lobule determines non-lateralized deficits which may contribute to neglect signs [24].

In the present study, line bisection was more sensitive than target cancellation. Similar results were reported in patients with Alzheimer's disease [25]. Simultagnosia and object recognition deficits, which may add noise to patients' performance on visual search tasks, can account for this finding. However, dissociations between line bisection and target cancellation have been previously reported in patients with focal lesions [26], and suggest that partially distinct neurocognitive systems are at work. For example, biased line bisection, which depends on dysfunction of the parietal lobe and of its connections with frontal regions [27], resulted from more posterior brain damage than impaired visual search [26].

The general lack of correlations between neglect severity and the number of years since PCA symptom onset suggests that, rather than a general consequence of late-stage PCA, neglect symptoms occur in certain PCA patients and not in others. Possibly this is because, in some PCA patients, bilateral atrophy can decrease the competitive interactions between the parietal lobes and contribute to lateralized neglect signs.

## Conclusion

Diagnosis of neglect has important implications for patient management, because of its dramatic clinical consequences on patients' everyday life[16]. For example, the presence of even mild degrees of neglect or visual extinction puts patients at risk of car accidents if they continue driving. Neglect also increases the risk of falls [28] and may contribute to spatial disorientation and wandering in patients with neurodegenerative conditions. Clinicians should consider the routine use of neglect tests such as line bisection and target cancellation as a cost-effective procedure to screen neurodegenerative patients [29].

## Competing interests

The authors declare that they have no competing interests.

## Authors' contributions

KA reviewed and collected medical, neuropsychological and neuroimaging data, and wrote the manuscript. DS was responsible for patients' neuropsychological assessment. MS and BD were responsible for patients' first neurological evaluation and their management. LCS helped to collect neuropsychological data. PB, LC, BD and MTS assembled the test battery, and MTS also helped to draft the manuscript. PB conceived the study, participated in its coordination and drafted the manuscript. All authors read and approved the final manuscript.

## Pre-publication history

The pre-publication history for this paper can be accessed here:

http://www.biomedcentral.com/1471-2377/10/68/prepub
